# Severe Pyodermia from Bromides

**Published:** 1911-06-17

**Authors:** 


### Medicine.

### SEVERE PYODERMIA FROM BROMIDES.

The fact that bromide of potassium frequently produces troublesome furuncles is familiar enough, but it is sometimes forgotten that instead of there being only a few scattered pustules which in themselves matter comparatively little the drug occasionally produces a very severe pyodermia which may be in itself a serious illness to the patient, and whose nature may not be immediately evident unless the possibility of the drug rashes is borne in mind. The five chief types of bromide rash are, first, ordinary disseminated furuncles; especially of the face and trunk, seldom of the limbs; secondly a generalised eczema; thirdly, localised aggregations of pustules with much induration like that of ordinary acne induratum; fourthly, an eruption like erythema nodosum but more persistent than the latter and not confined to the shins; fifthly, a generalised and almost confluent condition of acne-like pustules which might be termed acne-confluens. The latter appears in the form of oblong or roundish swellings, particularly on the lower extremities, of a rose or cherry-red colour, becoming yellowish by reason of millet-seed-like yellowish prominences upon them which are really acneiform pustules. The swellings have hard bases and are unaccompanied by swellings of the lymphatic glands or by feverish symptoms.

In a case of epilepsy, 8-grain doses of bromide of potassium were given three times a day and as the fits persisted the dose was increased to 10 grains three times a day. Notwithstanding this the fits persisted, though they abated in their frequency and severity, and by successive increases the bromide administered rose to 15, 20, and finally to 25 grains thrice daily. By this time the fits ceased to occur and all seemed to be going well, when, about a week after the largest doses were begun, boils began to appear upon the patient's legs and face. The eruption was not of the ordinary acne-like type, but might be described as resembling confluent chicken-pox or the vesicular stage of small-pox. The vesicles, however, instead of drying up and forming scabs, became aggregated together and the clusters spread at their margins, whilst upon the surface of the patches multiple points of pyodermia appeared. The patient developed pains in the head and a general feeling of illness, in addition to which, notwithstanding the continuance of the bromide in full doses, the epileptic fits recurred, and it was decided therefore to stop the drug. That the skin eruption was really due to the bromide was shown by the fact that it immediately began to improve, and also by the fact that when the drug was resumed on account of the continuance of the epilepsy the skin eruption occurred again with even greater severity than before. It was most extensive on the face and forehead and on the front and outer side of each leg from the knee to the ankle. On the face the affected skin was reddened and covered with light-brown crusts set closely together and each more or less circular in outline, these crusts being raised considerably above the surrounding red surface and varying in area from that of an ordinary pea to that of a sixpenny-piece; they were firm, flat, and adherent to such a degree that when attempts were made to remove them the underlying skin surface bled readily. The lesions upon the legs were in an even more acute state, the skin all round and between the actual spots of the eruption being of a vivid red colour, extremely tender, hot and painful, especially when the legs were not kept at absolute rest. The pains were chiefly of a burning and tingling type and they were worse at some times than at others. The elevations formed by the eruptions were very variable in size and less regular in shape than those upon the face. The smallest and most recently formed were about a quarter of an inch in diameter, consisting of a circular prominent bulging vesicle filled with a milk-like semi-fluid matter on a slightly elevated hardened base, which was surrounded by an acute red ring. Each vesicle seemed to have developed practically around the point of egress of a hair. The larger spots were flattened and covered either by moist exudation or with thick light-brown crusts, in either case surrounded by a dark red areola; if the crusts were removed numerous closely set yellowish-red foci of pus were exposed beneath it. When the progress of a single spot was watched from its commencement it was found to begin as a minute pimple, red, hot, tender, and painful. In a very short time a small conical yellowish-white vesicle appeared on its summit and in each case around a hair follicle; investigations showed that at least a part of the contents of the vesicle was fatty material derived from secretion of the sebaceous gland. If the vesicle was left alone it increased rapidly in size, became flatter and less tense, its contents becoming more fluid and purulent until finally by the running together of several such spots and the drying of the contents of the vesicles, the characteristic crusts with the multiple purulent foci beneath them were produced.

Fortunately so severe a degree of bromide pyodermia is rare, but sometimes it occurs and is mistaken for something else. The best cure for the condition is the omission of the bromide whenever this is possible, and it is noteworthy that the greatest immediate relief to the burning heat and pain is to be obtained by the use of a lotion containing bismuth together with decoction of poppies.

